# Metabolomic analysis-identified 2-hydroxybutyric acid might be a key metabolite of severe preeclampsia

**DOI:** 10.1515/biol-2022-0572

**Published:** 2023-02-28

**Authors:** Fang Wang, Lili Xu, Mingming Qi, Huimin Lai, Fanhua Zeng, Furong Liang, Qing Wen, Xihua Ma, Chan Zhang, Kaili Xie

**Affiliations:** Department of Obstetrics, Zhuzhou Central Hospital, Zhuzhou, 412007, China

**Keywords:** severe preeclampsia, liquid chromatograph mass spectrometer, metabolomic analysis, 2-hydroxybutyric acid

## Abstract

This study set out to determine the key metabolite changes underlying the pathophysiology of severe preeclampsia (PE) using metabolic analysis. We collected sera from 10 patients with severe PE and from 10 healthy pregnant women of the same trimester and analyzed them using liquid chromatography mass spectrometry. A total of 3,138 differential metabolites were screened, resulting in the identification of 124 differential metabolites. Kyoto encyclopedia of genes and genomes pathway analysis revealed that they were mainly enriched in the following metabolic pathways: central carbon metabolism in cancer; protein digestion and absorption; aminoacyl-transfer RNA biosynthesis; mineral absorption; alanine, aspartate, and glutamate metabolism; and prostate cancer. After analysis of 124 differential metabolites, 2-hydroxybutyric acid was found to be the most critical differential metabolite, and its use allowed the differentiation of women with severe PE from healthy pregnant women. In summary, our analysis revealed that 2-hydroxybutyric acid is a potential key metabolite for distinguishing severe PE from healthy controls and is also a marker for the early diagnosis of severe PE, thus allowing early intervention.

## 1 Introduction

Preeclampsia (PE) is defined as a new onset of hypertension during pregnancy after the 20th week [1], and its global prevalence is 8% [2]. Worldwide, PE and eclampsia are major causes of maternal and infant mortality [3]. In developing countries, it occurs at a rate of 1.8–16.7% [4] and causes 40–60% of maternal deaths [4]. Its rates have remained unchanged for decades, but the rates of severe PE have increased over recent decades [5]. Eclampsia can occur in patients with severe PE leading to symptoms of the nervous system [6] or hemolysis, elevated liver enzymes, low platelet count (HELLP) syndrome [7]. Depending on the clinical characteristics of a patient, PE can be classified as mild or severe [8]. Furthermore, it can be classified according to its time of clinical manifestation as “early-onset PE” (EOPE) in cases occurring before 34 weeks of pregnancy, or as “late-onset PE” (LOPE) in cases occurring after 34 weeks of pregnancy [9]. EOPE and LOPE might be more useful subclassifications [10]. The majority of the affected women suffers from PE at the late preterm or term stage, but about 12% suffers from PE that begins early (before 34 weeks of pregnancy) [11]. EOPE is the result of placental defects and deficiency of trophoblast invasion and normal spiral artery remodeling; LOPE, however, may result from interactions between the normal senescence of the placenta and a maternal genetic history of cardiovascular disease [12,13]. Blood pressure (BP) control is crucial during PE to prevent systemic complications [14]. Therefore, early diagnosis and early intervention in PE are particularly important.

There are many established risk factors for PE, such as nulliparity, advanced maternal age, overweight or obesity, chronic hypertension, diabetes, previous PE, family history of PE, and multiple pregnancy [15]; however, the exact causes of PE/eclampsia remain unclear [16]. Further research is needed concerning the cellular and molecular mechanisms of PE to improve the treatment of PE patients [17].

The study of metabonomics and metabolomics involves the use of accurate metabonomic (and/or metabolomic) analyses of metabolic changes occurring in cells, tissues, and whole organisms [18]. It is part of the “omics cascade” together with genomics, transcriptomics, and proteomics [19] and one of the many “-omics” technologies that are currently being developed [20]. Metabolomics, or metabonomics, primarily involves the elucidation of the end products in a specific organism or a cell [21], and it is the “ultimate” tool in the “omics chain,” as it is the closest to the phenotype [22]. It can be divided into two categories: untargeted and targeted [23]. While the former also known as discovery metabolomics, which is a global analysis of different metabolomics between control and experimental groups, the latter focuses on the analysis of specific metabolic clusters associated with certain metabolic pathways [20].

2-Hydroxybutyric acid is elevated in many diseases and has some diagnostic values. In cancer, its levels have been elevated in a mouse model of colon carcinogenesis induced by azoxymethane/dextran sodium sulfate [24]; furthermore, nuclear lactate dehydrogenase A induces its production from reactive oxygen species and promotes human papilloma virus-induced cervical tumor growth [25]; moreover, patients in the initial diagnostic stage of acute myeloid leukemia can be identified by 2-hydroxybutyric acid [26]. In pneumonia diseases, compared to healthy controls, 2-hydroxybutyric acid was found to be enriched in COVID-19 patients and COVID-like patients and remained at higher levels after discharge [27]; meanwhile, elevated dehydrogenase can be an independent prognostic factor for death in hospitalized COVID-19 patients [28]; it also has a diagnostic value in community-acquired pneumonia [29]. 2-Hydroxybutyric acid has been more deeply studied in diabetes than in any other disease, and high levels of plasma are a good predictor of type 2 diabetes [30]. Furthermore, it can be used in the following circumstances: as a biomarker of insulin resistance; for disease tracking throughout the treatment of insulin resistance [31]; as a predictive marker for impaired glucose tolerance without the need for a glucose tolerance test [32]; along with branched-chain amino acids, it can predict worsening glycemic control in adolescents [33]; it has high values in the sera of patients with isolated postchallenge diabetes compared to normal subjects [34]; and its levels decrease significantly after laparoscopic sleeve gastrectomy in morbidly obese patients [35]. It also have some diagnostic values in other diseases: it is significantly higher in the blood of pregnant women carrying trisomy 21 fetuses than in healthy pregnant women [36]; urinary 2-hydroxybutyric acid predicts the development of acute kidney injury in presurgical samples [37]; and its levels are elevated in patients with a major depressive disorder [38].

In this study, we collected sera from 10 patients with severe PE and 10 healthy pregnant women of the same trimester and analyzed them using liquid chromatograph mass spectrometry (LC-MS), with a view to identifying the key metabolites of the former pathogenesis and providing new indicators for early diagnosis.

## 2 Materials and methods

### 2.1 Study population

All samples were obtained from the Obstetrics Department of Zhuzhou Central Hospital and were divided into a normal control group (10 normotensive pregnant women) and a severe PE group (*n* = 10). The basic diagnostic criteria for PE are as follows: BP ≥140/90 mmHg, and urine protein ≥0.3 mg/24 h. Severe PE is diagnosed on the basis of the diagnostic criteria for PE with any of the following conditions present: systolic BP ≥160 mmHg, or diastolic BP ≥110 mmHg, or other manifestations of a multisystem disorder (e.g., severe proteinuria, thrombocytopenia, impaired liver function, severe persistent right upper quadrant or epigastric pain, renal insufficiency, pulmonary edema, or new-onset headache).


**Informed consent:** Informed consent has been obtained from all individuals included in this study.
**Ethical approval:** The research related to human use has been complied with all the relevant national regulations, institutional policies and in accordance with the tenets of the Helsinki Declaration, and has been approved by the Institutional Research Ethics Board of Zhuzhou Central Hospital (reference number 20180334).

### 2.2 Sample processing

A peripheral blood sample (10 mL) was taken from each participant. All samples were centrifuged at 2,000 rpm for 10 min at room temperature using a centrifuge. Afterward, the supernatant was stored in a refrigerator at −80°C.

### 2.3 Spectroscopy

All samples were thawed at 4°C (insufficient samples were reduced to an equal scale); 100 µL of each sample was transferred into 2 mL centrifuge tubes (samples with a sample size of <50 µL were extracted by half of the experimental system, but the resolution system remained unchanged); 400 µL of methanol (−20°C) was added to each tube and vortexed for 60 s; the mixture was centrifuged at 4°C for 10 min at 12,000 rpm, and then the supernatant was transferred from each sample into another 2 mL centrifuge tube. Samples were concentrated to dry in a vacuum and subsequently dissolved with 150 µL 2-chlorobenzalanine (4 ppm) 80% methanol solution, and the supernatant was filtered through a 0.22 µm membrane to obtain the prepared samples for gas chromatography mass spectrometry (GC-MS). For quality control (QC), 20 µL subsamples were taken (QC samples were used to monitor deviations of the analytical results from these pool mixtures and compare them to the errors caused by the analytical instrument itself). The remainder of the samples were used for LC-MS detection

### 2.4 Chromatography and mass spectrometry conditions

Chromatographic separation was performed with an ACQUITY UPLC^®^ HSS T3 (150 mm × 2.1 mm, 1.8 µm, Waters) column maintained at 40°C. The temperature of the autosampler was 8°C. Gradient elution of analytes was carried out with 0.1% formic acid in water and 0.1% formic acid in acetonitrile or 5 mM ammonium formate in water and acetonitrile at a flow rate of 0.25 mL/min. Injection of 2 μL of each sample was performed after equilibration. An increasing linear gradient of solvent B (v/v) was used as follows: 0–1 min, 2% B/D; 1–9 min, 2–50% B/D; 9–12 min, 50–98% B/D; 12–13.5 min, 98% B/D; 13.5–14 min, 98–2% B/D; 14–20 min, 2% D-positive model (14–17 min, 2% B-negative model).

The electrospray ionization multistage mass spectrometry experiments were used with a spray voltage of 3.5 and −2.5 kV in positive and negative modes, respectively. Sheath gas and auxiliary gas were set at 30 and 10 arbitrary units, respectively, while the capillary temperature was 325°C. The Orbitrap analyzer scanned over a mass range of *m*/*z* 81–1,000 for a full scan at a mass resolution of 70,000. Data-dependent acquisition MS/MS experiments were performed with a higher energy collisional dissociation scan. The normalized collision energy was 30 eV. Dynamic exclusion was implemented to remove some unnecessary information in MS/MS spectra.

### 2.5 Multivariate statistical analysis

The data were analyzed using SIMCA-P (v13.0) [35] software and the R language ropls [39] package. The main methods of analysis included principal component analysis (PCA), partial least squares-discriminant analysis (PLS-DA), and orthogonal partial least squares discriminant analysis (OPLS-DA) [40].

Unsupervised analysis (e.g., PCA) does not ignore within-group errors, eliminates random errors that are not relevant to the purpose of the study, focuses too much on details, and neglects the overall picture and patterns, and is ultimately detrimental to the detection of between-group differences and differential compounds. In such cases, it is necessary to use prior knowledge of the sample to further focus the data analysis on the aspect being studied, using a supervised analysis such as PLS-DA. OPLS-DA, another commonly used method in metabolomics data analysis, is an extension of PLS-DA. Compared to the PLS-DA, this method can effectively reduce the complexity of the model and enhance the explanatory power of the model without reducing the predictive power, thus maximizing the differences between groups.

## 3 Analysis and identification of metabolites

### 3.1 Differential metabolite screening

Metabolites are screened to identify differential metabolites (biomarkers); the relevant conditions are as follows: *p*-value ≤0.05 + VIP (variable importance for the projection) ≥1.

### 3.2 Identification of metabolites

Metabolite identification was first confirmed on the basis of precise molecular weights (molecular weight error <30 ppm), followed by confirmation of annotation against the Metlin (http://metlin.scripps.edu) and MoNA (https://mona.fiehnlab.ucdavis.edu//) databases based on MS/MS fragmentation patterns to identify the final metabolites.

### 3.3 Network analysis

Metscape [41], a Cytoscape plug-in (v.3.9.0) [42], was used for the metabolic network analysis and data visualization.

### 3.4 Kyoto encyclopedia of genes and genomes (KEGG) analysis

MetPA is part of metaboanalyst (www.metaboanalyst.ca) and is based on the KEGG metabolic pathway. The MetPA database identifies possible bioturbated metabolic pathways through metabolic pathway enrichment and topology analysis, and thus analyzes the metabolic pathways of metabolites. The MetPA database allows the analysis of metabolic pathways associated with two sets of differential metabolites, using a hypergeometric test as the data analysis algorithm, and relative-betweeness centrality for pathway topology.

## 4 Results

### 4.1 Subject characteristics

Ten healthy pregnant women of the same trimester and 10 pregnant women with severe PE were included in this study. There were no significant differences between the two groups when subject characteristics such as age (31.6 ± 5.77) or week of gestation (32.1 ± 1.72 W) were taken into account. However, there were significant differences in BP, gestational week at termination, neonatal Apgar score, and neonatal weight. Table 1 shows the clinical features of the 10 severe PE cases.

**Table 1:** Severe PE clinical features

NO	Age (years)	Gestational weeks	Systolic pressure (mmHg)	Edema	24 h urine protein (g/L)	Albumin	Ultrasound of pleural and peritoneal effusion	Time to terminate pregnancy	Neonatal Apgar Scores	Neonatal weight (g)	Echocard-iography
1	25	33 + 3	116–151	Normal	11.120	22.6 g/L	Normal	34 weeks	8–10	1,600	Normal
2	32	29 + 5	109–161	(+)	0.603	Normal	Normal	30 + 6	9–10	1,270	Normal
3*	31	31 + 5	100–139	(+)	0.378	25.2 g/L	Normal	35 + 1	9–10	2,160 and 2,420 (twins)	Normal
4	29	32 + 3	120–160	Normal	1.229	Normal	Normal	Leave hospital	Leave hospital	Leave hospital	Normal
5	33	31 + 2	150–175	Normal	Leave hospital	Leave hospital	Normal	Leave hospital	Leave hospital	Leave hospital	Normal
6	45	32 + 6	107–166	Normal	5.292	24 g/L	Normal	32 + 6	7–10	1,250	Normal
7	31	29	130–170	Normal	6.134	Normal	Normal	29 + 2	0	1,050	＜5 mL
8	24	32 + 6	122–161	(++++)	4.772	27.2 g/L	Pleural effusion	33 + 2	9–10	1,640	Normal
9	34	33 + 3	110–147	(++)	0.454	25.1 g/L	Pleural effusion	33 + 3	8–10	1,720 and 1,790 (twins)	Normal
10	34	34 + 3	118–175	(+++)	1.742	26.5 g/L	Pleural effusion	34 + 6	8–10	1,920 and 1,890 (twins)	Normal

Note: NO 7, The patient requested induction of labor, rivanol amniotic injection at 29 + 2 weeks, neonatal death. No 3 had a diastolic BP of 110 mmHg. *Had a diastolic BP of 110 mmHg.

### 4.2 Multivariate analysis

**Figure 1:**
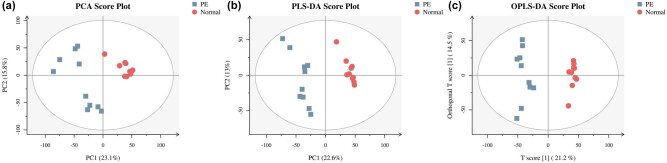
(a) PCA, (b) PLS-DA, and (c) OPLS-DA of the severe EOPE and normal groups.

In this study, serum samples from the 10 healthy pregnant women and 10 severe PE patients were analyzed with a metabolomics approach, using GC-MS followed by the multivariate data analysis by PCA, PLS-DA, and OPLS-DA (Table 2). Initial unsupervised PCA (Figure 1a) and supervised PCA (Figure 1b and c) showed a clear separation of metabolites between the normal and severe EOPE groups.

**Table 2:** Multivariate data analysis result

PE vs Normal	Pre	R2X (cum)	R2Y (cum)	Q2 (cum)
PCA	4	0.511		
PLS-DA	3	0.432	0.998	0.962
OPLS-DA	1 + 1 + 0	0.356	0.973	0.895

### 4.3 Identified metabolites

**Figure 2:**
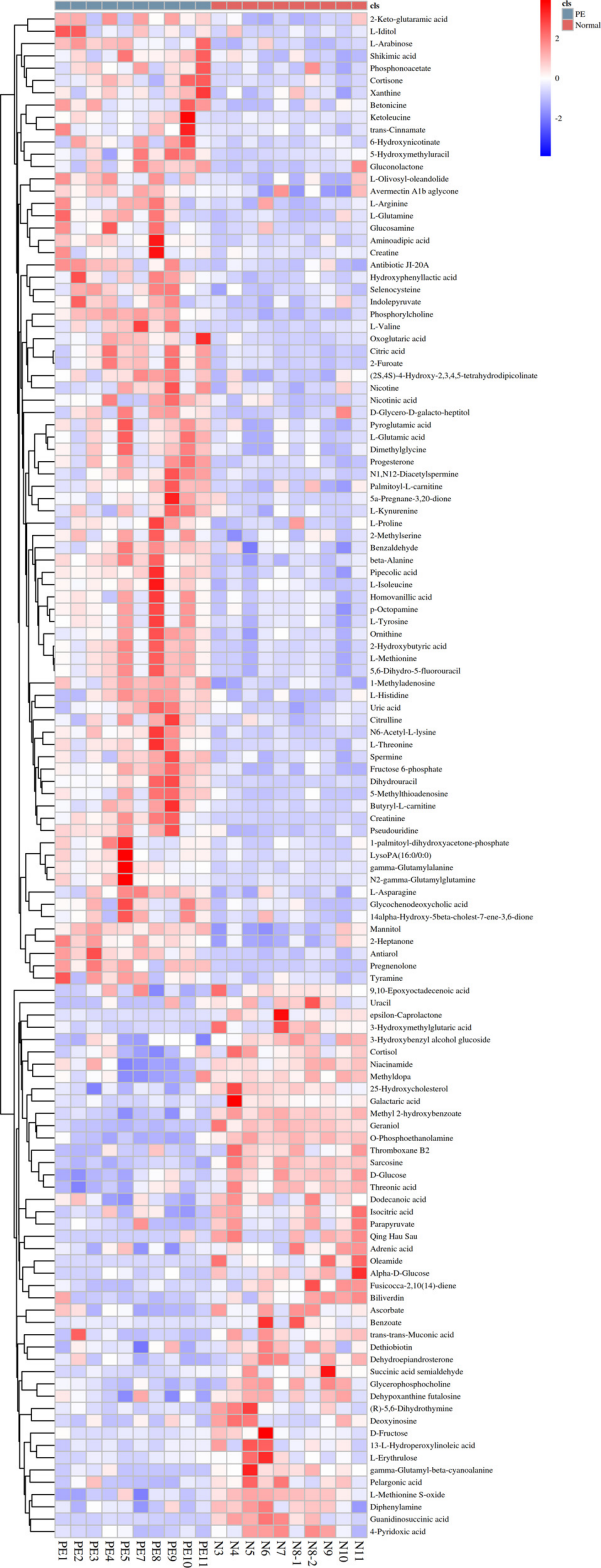
Heatmap with all the significant metabolites.

**Figure 3:**
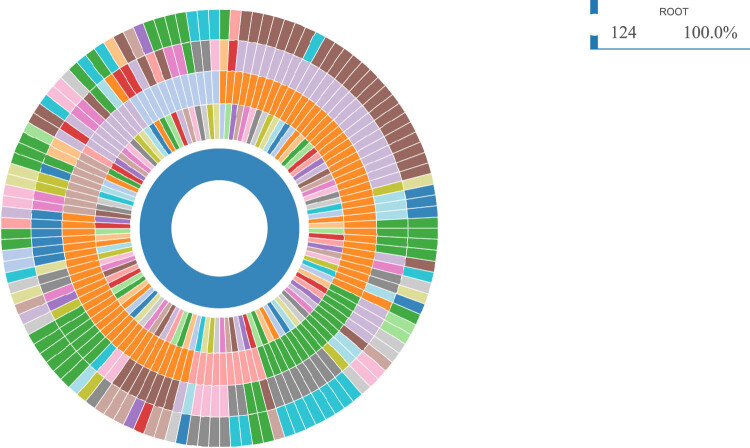
Classification of identified differential metabolites. The differential metabolites located on metabolism of amino acids, carbohydrates, cofactors and vitamins, lipids, nucleotides, and peptides.

In the GC-MS analysis, 3138 differential metabolites were screened and 124 metabolites (Figure 2) were eventually identified. Of the 124 identified differential metabolites, 45 downregulated products and 79 upregulated products were included. The classification of the identified differential metabolites are summarized in Figure 3; these were mainly located on metabolism of amino acids, carbohydrates, cofactors and vitamins, lipids, nucleotides, and peptides.

### 4.4 Network analysis

**Figure 4:**
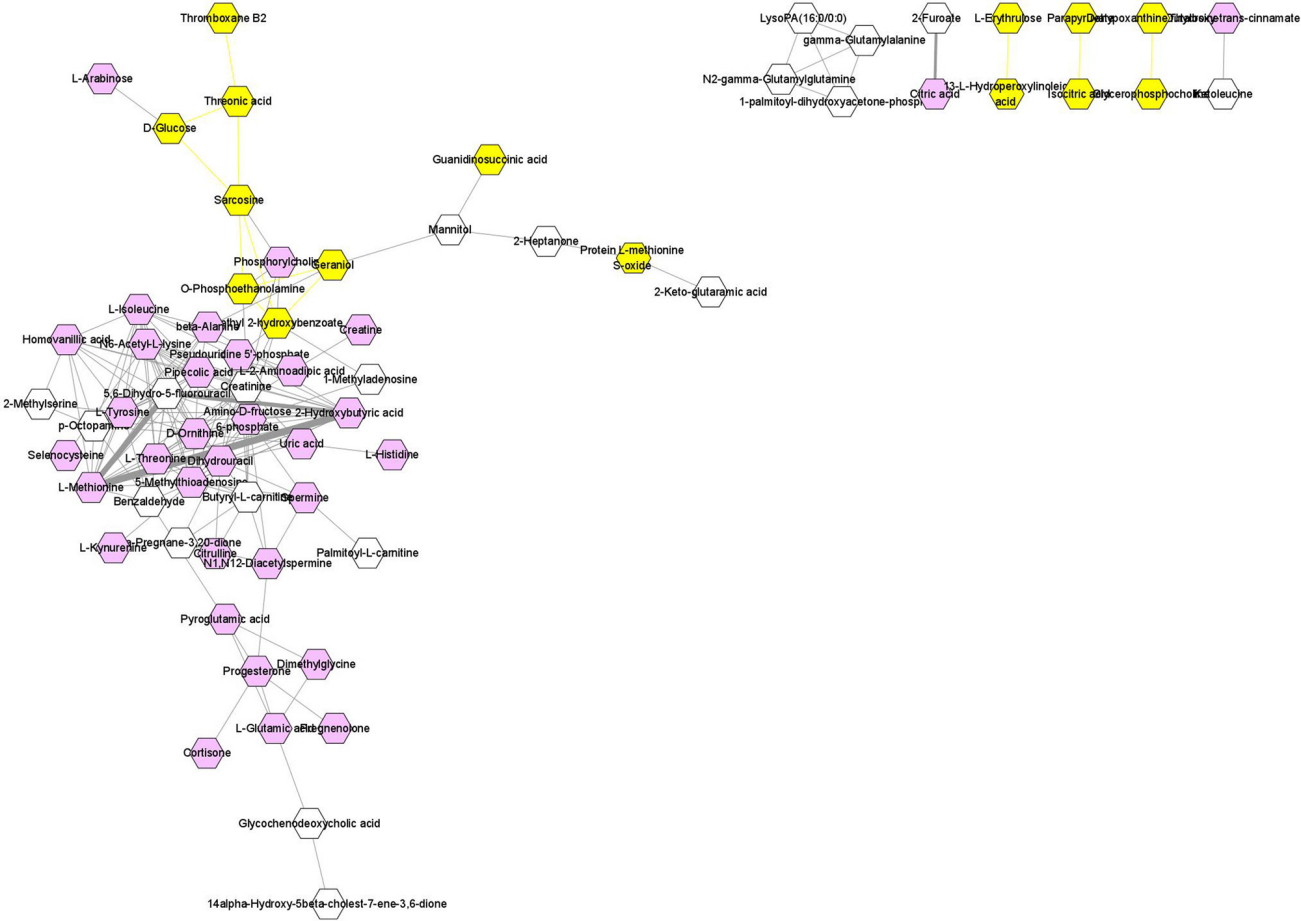
Network analysis of identified differential metabolites. 2-Hydroxybutyric acid was identified as the key metabolite in a network analysis based on VIP and FDR values.

**Figure 5:**
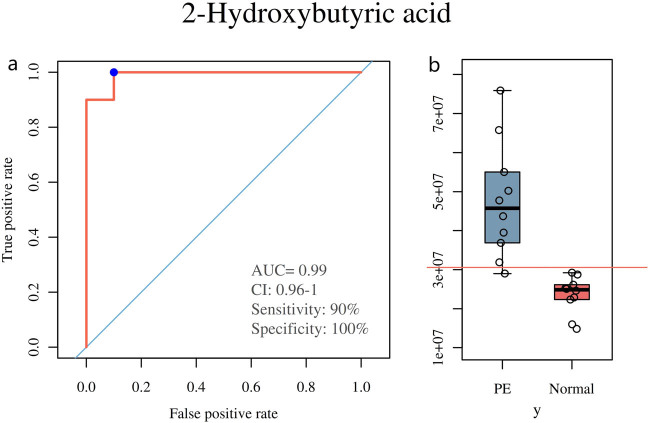
ROC plot of 2-hydroxybutyric acid and the concentrations of 2-hydroxybutyric acid in PE group and normal group. (a) There was an AUC of 0.99. (b) The concentrations of 2-hydroxybutyric acid is 47559849.097 ± 14839964.376 in the PE group and 23509331.240 ± 4802006.390 in the normal group.

By building an association-based network, we found that 2-hydroxybutyric acid was important within this metabolic network (Figure 4). Furthermore, the results of the differential metabolite analysis showed that 2-hydroxybutyric acid differed significantly between preeclamptic and normal pregnant women (VIP = 1.670, *p*-value <0.05, false discovery rate (FDR) = 0.006, log FC = 1.016). Meanwhile, the results of analysis of variance for l-threonine and 5,6-dihydro-5-fluorouracil were VIP = 1.695, *p*-value <0.05, FDR = 0.022, log FC = 1.036; and VIP = 1.654, *p*-value <0.05, FDR = 0.006, log FC = 1.020, respectively. In the ROC plot (Figure 5), there was an area under the curve (AUC) of 0.99, indicating a high level of accuracy (high accuracy = AUC >0.9).

### 4.5 KEGG analysis

**Figure 6:**
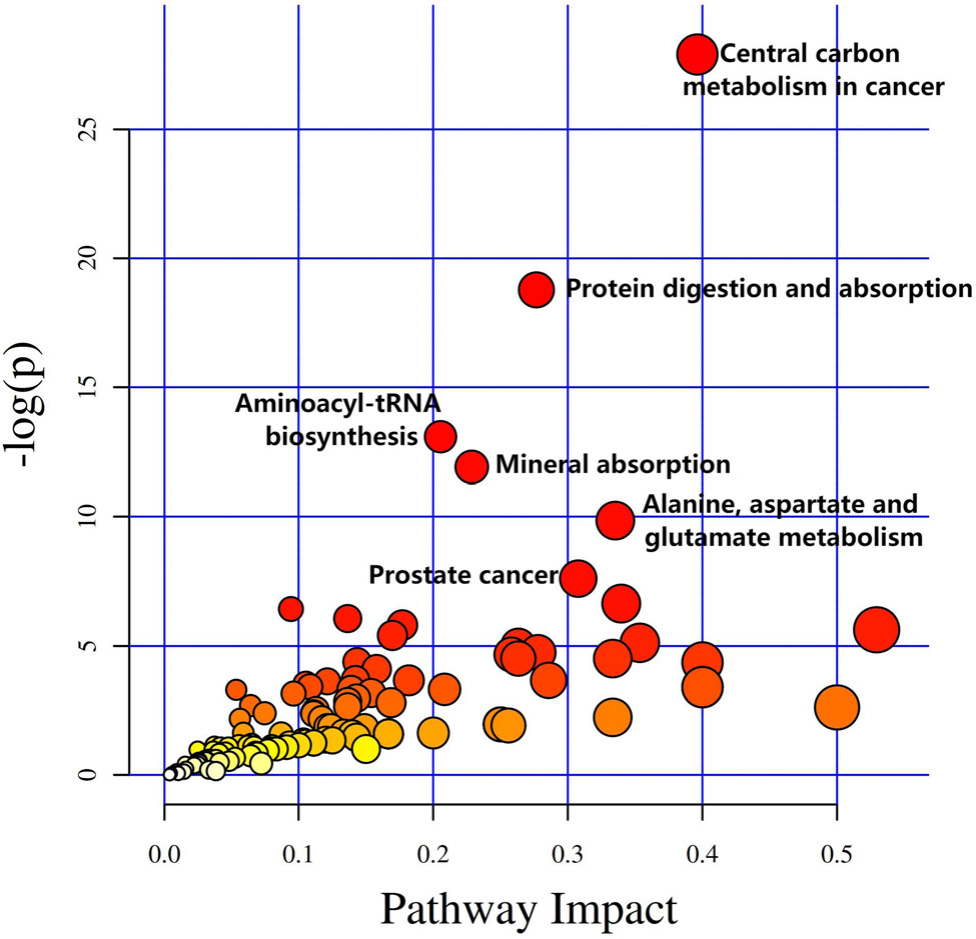
KEGG pathway analysis of identified differential metabolites. Significantly enriched pathways include central carbon metabolism in cancer (37 differential metabolites); protein digestion and absorption (47 differential metabolites); aminoacyl-tRNA biosynthesis (52 differential metabolites); mineral absorption (29 differential metabolites); alanine, aspartate, and glutamate metabolism (28 differential metabolites); prostate cancer (11 differential metabolites) (FDR <0.05). The size of the circle represents the number of differential metabolites that are enriched in this pathway. −log(*p*): Negative values for the natural logarithm of the *p*-value. Impact: Metabolic pathway impact values.

The final results of the differential metabolite KEGG pathway enrichment analysis are shown in Figure 6. Significantly enriched pathways included central carbon metabolism in cancer, protein digestion and absorption, aminoacyl-transfer RNA (tRNA) biosynthesis, mineral absorption, alanine, aspartate and glutamate metabolism, and prostate cancer (FDR <0.05).

## 5 Discussion

In this study, we analyzed the metabolites in the sera of 10 severe PE cases and 10 healthy pregnant women. A total of 3,138 differential metabolites were screened, resulting in the identification of 124 differential metabolites. After analysis of 124 differential metabolites, 2-hydroxybutyric acid was found to be the more critical differential metabolite; its presence clearly distinguished between severe PE and healthy pregnant women. The analysis of the KEGG pathway revealed that the metabolites were mainly enriched in the following metabolic pathways: central carbon metabolism in cancer; protein digestion and absorption; aminoacyl-tRNA biosynthesis; mineral absorption; alanine, aspartate, and glutamate metabolism; prostate cancer.

2-Hydroxybutyric acid is predominantly produced during the metabolism of l-threonine or the synthesis of glutathione and may be elevated by oxidative stress or the detoxification of exogenous substances in the liver [43]. It has been previously demonstrated that 2-hydroxybutyric acid, as a component of a metabolite-only model, can predict the EOPE [44]. Our study also found that this metabolite played an important role in severe PE; however, we used a different approach.

Two of the KEGG enrichment analyses differential metabolites that we studied were associated with cancer. The other four KEGG pathways were all found to be associated with severe PE. The differential mRNAs between the preeclamptic and normal groups were also found to be enriched in the protein digestion and absorption pathways [45]. Thus, previous studies have found that l-arginine supplementation can be used to treat individuals with PE [46]. Harville et al. demonstrated that aminoacyl-tRNA biosynthesis is associated with hypertensive disorders of pregnancy [47]. Furthermore, the relationship between minerals and PE has been relatively well studied. Serum selenium levels have been shown to be associated with PE in several studies [48–51]; however, amniotic fluid selenium status has been shown to be uncorrelated with PE or preterm delivery [52,53]. On the other hand, Enebe et al. found that low levels of antioxidant trace elements, for example, selenium, copper, and magnesium, can promote the incidence of PE [54], and other studies have found that mineral and vitamin supplementation can reduce the incidence [55,56]. These findings illustrate the impact of mineral absorption pathways on PE. To our knowledge, there is no literature addressing the relationship between the alanine, aspartate, and glutamate metabolism pathway and PE. However, a proportion of patients with PE do have abnormal liver function.

In summary, our analysis revealed that 2-hydroxybutyric acid might be a key metabolite for distinguishing severe PE from normal controls and is potentially a marker for the early diagnosis of severe PE, thus providing a basis for early detection and intervention.
